# Investigating the association of the plasma lipidomic profile with cognitive performance and genetic risk in the PsyCourse study

**DOI:** 10.1038/s41398-025-03323-5

**Published:** 2025-03-28

**Authors:** Mojtaba Oraki Kohshour, Sergi Papiol, Anna Tkachev, Elena Stekolshchikova, Kristina Adorjan, Monika Budde, Urs Heilbronner, Maria Heilbronner, Janos L. Kalman, Daniela Reich-Erkelenz, Sabrina K. Schaupp, Fanny Senner, Thomas Vogl, Jens Wiltfang, Eva Z. Reininghaus, Georg Juckel, Udo Dannlowski, Andreas J. Fallgatter, Carsten Spitzer, Max Schmauß, Martin von Hagen, Peter Falkai, Philipp Khaitovich, Thomas G. Schulze, Eva C. Schulte

**Affiliations:** 1 Institute of Psychiatric Phenomics and Genomics (IPPG), LMU University Hospital, LMU Munich, Munich, 80336 Germany; 2 Department of Immunology, Faculty of Medicine, Ahvaz Jundishapur University of Medical Sciences, Ahvaz, Iran; 3 Max Planck Institute of Psychiatry, Munich, 80804 Germany; 4 Vladimir Zelman Center for Neurobiology and Brain Rehabilitation, Skolkovo Institute of Science and Technology, Moscow, 121205 Russia; 5 University Hospital of Psychiatry and Psychotherapy, University of Bern, Bern, Switzerland; 6 Department of Psychiatry and Psychotherapy, LMU University Hospital, LMU Munich, Munich, 80336 Germany; 7 Centres for Psychiatry Suedwuerttemberg, Ravensburg, 88214 Germany; 8 Department of Psychiatry and Psychotherapy, University Medical Center Göttingen, Göttingen, 37075 Germany; 9 German Center for Neurodegenerative Diseases (DZNE), Göttingen, 37075 Germany; 10 Neurosciences and Signaling Group, Institute of Biomedicine (iBiMED), Department of Medical Sciences, University of Aveiro, Aveiro, Portugal; 11 Division of Psychiatry and Psychotherapeutic Medicine, Research Unit for Bipolar Affective Disorder, Medical University of Graz, Graz, 8036 Austria; 12 Department of Psychiatry, Ruhr University Bochum, LWL University Hospital, Bochum, 44791 Germany; 13 Institute for Translational Psychiatry, University of Münster, Münster, 48149 Germany; 14 Department of Psychiatry and Psychotherapy, Tübingen Center for Mental Health (TüCMH), University of Tübingen, Tübingen, 72076 Germany; 15 German Center for Mental Health (DZPG), partner site Tübingen, Tübingen, 72076 Germany; 16 Department of Psychosomatic Medicine and Psychotherapy, University Medical Center Rostock, Rostock, 18147 Germany; 17 Clinic for Psychiatry, Psychotherapy and Psychosomatics, Augsburg University, Medical Faculty, Bezirkskrankenhaus Augsburg, Augsburg, 86156 Germany; 18 Clinic for Psychiatry and Psychotherapy, Clinical Center Werra-Meißner, Eschwege, 37269 Germany; 19 German Center for Mental Health (DZPG), partner site Munich/Augsburg, 80336, Munich, Germany; 20 Department of Psychiatry and Behavioral Sciences, Norton College of Medicine, SUNY Upstate Medical University, Syracuse, NY USA; 21 Department of Psychiatry and Behavioral Sciences, Johns Hopkins University School of Medicine, Baltimore, MD USA; 22 Department of Psychiatry and Psychotherapy, Faculty of Medicine and University Hospital Bonn, University of Bonn, Bonn, 53127 Germany; 23 Institute of Human Genetics, Faculty of Medicine and University Hospital Bonn, University of Bonn, Bonn, 53127 Germany

**Keywords:** Molecular neuroscience, Learning and memory

## Abstract

Although lipid biology may play a key role in the pathophysiology of mental health disorders such as schizophrenia (SCZ) and bipolar disorder (BD), the nature of this interplay and how it could shape phenotypic presentation, including cognitive performance is still incompletely understood. To address this question, we analyzed the association of plasma level of different lipid species with cognitive performance in the transdiagnostic PsyCourse Study. Plasma lipidomic profiles of 623 individuals (188 SCZ, 243 BD, 192 healthy controls) belonging to the PsyCourse Study were assessed using liquid chromatography and untargeted mass spectrometry. The association between 364 annotated lipid species from 16 lipid classes and six cognitive tests was evaluated. Likewise, the association of polygenic risk scores (PRS) for SCZ, BD, executive function (EF), and educational attainment (EA) with lipid plasma levels were also investigated. In the regression analysis, three lipid species belonging to phosphatidylethanolamine plasmalogen and one belonging to ceramide class showed significant negative association with Digit-Symbol test scores. Lipid class-based enrichment analysis in LipidR replicated the significance of the phosphatidylethanolamines class for the Digit-Symbol test, which evaluates the processing speed in cognitive tasks. Polygenic load for SCZ, BD, EF, or EA was not associated with lipid levels. Our findings suggest a link between lipids and cognitive performance independent of mental health disorders. Still, independent replication is warranted to better understand if phosphatidylethanolamines could represent an actionable pharmacologic target to tackle cognitive dysfunction, an important unmet clinical need that affects long-term functional outcomes in individuals with severe mental health disorders.

## Introduction

Schizophrenia (SCZ) and bipolar disorder (BD) are severe and chronic mental disorders with highly polygenic architecture and heterogeneous symptoms [1, 2]. Heritability estimates range around 60 to 80% and the global lifetime prevalence has been reported at around 0.8% for SCZ and 2–3% for BD [1, 3, 4]. The exact etiology of these major mental health disorders is yet unknown and while their symptoms overlap, their diagnostic criteria still are based on clinical evaluations of symptoms without objective markers [5, 6].

Given the association between plasma lipid profiles and clinical traits, lipid biology may have important functions in the pathophysiology of SCZ and BD [2, 7–9]. Lipids make up more than half of the brain’s dry weight, and myelin sheaths make up about 80% of all brain lipids [1]. In addition to a potential role as biomarkers, lipids and lipid intermediates hold important yet under-studied roles in brain structure and function [10]. More than 10% of prefrontal cortex (PFC) lipids in individuals with SCZ are significantly different from healthy controls [9]. Also, individuals at high risk of developing SCZ and BD exhibit abnormalities compatible with lipid dysregulation such as myelin dysfunction in the PFC that could lead to functional and cognitive impairments [1, 11, 12]. In our most recent study, a multi-cohort case-control study that included the PsyCourse Study, plasma lipid abnormalities were transdiagnostically and transethnically linked to diagnoses, such as SCZ, BD, and major depressive disorder (MDD) [13].

Cognitive dysfunction is among the most disabling symptoms of SCZ and BD and is difficult to treat with the commonly used pharmacologic regimes [14, 15]. Consequently, it has important impacts on long-term functional outcomes [15, 16]. Although the relationship between circulating lipids and cognitive performance is complex, disruptions in lipid homeostasis and clinical dyslipidemia are generally contributors to changes in cognitive performance in mental health disorders [17, 18]. While the relationship between clinical lipids, such as cholesterol and cognitive performance has been investigated in some depth, little is known about the role of the vast rest of the lipidome –i.e., non-clinically tested lipid species– in this context.

The fact that in neurotypical individuals, brain lipidomic profiles have been shown to be brain-region- and brain-cell type-specific and related to functional connectivity [19], argues for a likely role for brain lipidomic profiles and brain lipids well beyond those that are commonly assessed in clinical contexts to be relevant to cognitive processes. Several neurodevelopmental traits have been associated with specific dyslipidemia, e.g., autism spectrum disorder with decreased linoleic acid, intelligence quotient/developmental quotient composite score with increased arachidonic acid and sleep disturbances with decreased docosahexanoic acid and arachidonic acid [20]. In addition, previous studies in the field of neurodegenerative disorders such as Alzheimer’s diseases (AD) have demonstrated that links between the lipid profile and cognitive symptoms/changes may exist [21, 22]. It has been suggested that plasma lipid levels could differentiate two early AD subgroups with varying cognitive performance [23]. In this vein, a panel of plasma lipids (17-lipid signature) comprising acylcarnitines, sterol lipids, sphingolipids, and phospholipids, has been suggested to be positively associated with the Alzheimer Disease Assessment Scale–13‐item cognitive subscale for cognitive performance [21].

Regarding SCZ and BD, both central and peripheral abnormalities in lipidome composition and metabolism have been described that might affect cognitive performance [1, 5]. Individuals with SCZ presenting with metabolic syndrome and clinical dyslipidemia exhibit higher cognitive impairments (lower cognitive domain scores on tests measuring processing speed, attention/vigilance, working memory and problem solving/reasoning) [18, 24]. Changes in blood cholesterol, apolipoprotein A1, and apolipoprotein B levels in individuals with SCZ, and blood high-density lipoprotein and triglyceride levels in individuals with BD, have been associated with cognitive function especially as measured on composite memory-related scales (e.g., the Mini Mental State Examination (MMSE) or the Repeatable Battery for the Assessment of Neuropsychological Status (RBANS)) [17, 25–28].

Furthermore, shared genetic determinants of severe mental health disorders and lipid metabolism have been previously identified and genetic factors are known to determine peripheral lipid levels to a large extent [20, 29–31]. In psychiatry, dyslipidemia is a significant health concern, and it has been documented that lipid metabolism-related genes exhibit enrichment of genetic variants linked to certain mental health disorders [29, 32, 33]. Over 1000 genetic loci have been linked to blood lipid concentrations and lipid class heritability’s range from 0.2 to 0.5 [31, 34–39]. Polygenic complex traits, such as metabolic syndrome and mental health disorders, share several genetic risk loci [32] and genome-wide association studies (GWAS) have revealed an overlap between susceptibility loci for SCZ and BD and genes involved in the regulation of lipid levels [2].

Accordingly, the aim of the study presented herein was to explore the association of plasma lipid levels with cognitive performance by utilizing untargeted liquid chromatography-mass spectrometry (LC-MS)-based profiling approach in the PsyCourse Study. We also investigated if genetic liability in the form of polygenic risk scores (PRS) for SCZ (PRC-SCZ), BD (PRS-BD), executive function (PRS-EF), and educational attainment (PRS-EA; which can account for a portion of cognitive function in individuals with mental health disorders) [40] influence the lipid levels in our study. Compared to previous approaches, our current study vastly expands the range of mental health diagnoses, the depth of cognitive phenotyping, and the breadth of analyzed lipidomic features and addresses the role of genetic factors in the interplay between lipidomics and cognition. Yet, it still keeps a narrow focus on lipidomics and cognitive performance in mental health disorders.

## Subjects and methods

### Participants

A total of 623 individuals from the PsyCourse Study, all participants for whom plasma lipidomic data were available, were included in this investigation, among them 188 and 243 individuals with SCZ and BD, respectively, who had been diagnosed using DSM-IV criteria, as well as 192 healthy controls without a mental health diagnosis. The PsyCourse Study (www.psycourse.de) is a German/Austrian longitudinal cohort study, in which biomaterials and detailed phenotypic data from 1320 individuals with a range of mental health disorders and 466 individuals without a mental health diagnosis have been collected. The control group did not include any individuals with neurological diseases affecting the central nervous system, such as mental health disorders, epilepsy, stroke, multiple sclerosis, dementia, and structural brain impairments, or severe somatic comorbidities. The current analyses were based on version 5.0 of the PsyCourse dataset [41]. Written informed consent was obtained from each participant. The study was approved by the University Hospital Munich’s ethical committee (Project number: 17-13) and the other study sites [42], and was carried out in accordance with the Declaration of Helsinki.

### Cognitive performance assessment

A cognitive testing battery consisting of Trail-Making Test part A (TMT-A) and B (TMT-B) [43–45], Verbal Digit Span forward (DGT-SP-FRW) and backward (DGT-SP-BCK) [46], Digit-Symbol (DG-SYM) [47], and Multiple-choice Vocabulary Intelligence (Deutsch: Mehrfachwahl–Wortschatz–Intelligenz [MWT-B]) [48, 49] tests were administered by trained raters. The cognitive tests that provide scores (applied to our analyses), used to interpret cognitive performance are briefly explained in Table 1 [40–42] and detailed additional information on the tests can be obtained elsewhere [40–49]. Plasma sampling and cognitive testing were performed on the same day.

**Table 1:** Description of the tests assessed in the cognitive performance testing battery in the PsyCourse Study.

Name	Cognitive Domain	Description	Interpretation
TMT-A	Psychomotor speed (and to a lesser degree executive function)	Randomly distributed numbers need to be connected in ascending order in a timely manner (“1-2-3-4…”)	Higher values indicate poorer test performance
TMT-B	Executive function	Randomly distributed numbers and symbols need to be connected alternatingly and in ascending order in a timely manner (“1-A-2-B-3-C…”)	Higher values indicate poorer test performance
DGT-SP-FRW	Short-term memory	The interviewer reads increasingly longer strings of digits and asks the participant to repeat them	Lower values indicate poorer test performance
DGT-SP-BCK	Working memory	The interviewer reads increasingly longer strings of digits and asks the participant to repeat them backwards	Lower values indicate poorer test performance
DG-SYM	Processing speed (and to a lesser extend psychomotor speed)	Participants are asked to fill in a symbol that corresponds to a number by using a given number-symbol key as quickly as possible	Lower values indicate poorer test performance
MWT-B	Crystallized intelligence	Participants have to select the single existing German word in 37 sets of five “words” each	Lower values indicate poorer test performance

*TMT-A* trail-making test part A, *TMT-B* trail-making test part B, *DGT-SP-FRW* verbal digit span forward, *DGT-SP-BCK* verbal digit span backward, *DG-SYM* digit-symbol, *MWT-B* multiple-choice vocabulary intelligence.

### Lipid quantification

Plasma sample collection and lipid quantification were carried out as previously reported in Tkachev et al. [13]. In brief, non-fasting plasma samples were collected between 2012 and 2016 and analyzed between 2018 and 2020. Liquid chromatography coupled with untargeted mass spectrometry (LC-MS) consisted of a Waters Acquity UPLC system (Waters, Manchester, UK) and a Q Exactive orbitrap mass spectrometer (Thermo Fisher Scientific, USA) equipped with a heated electro-spray ionization (HESI) probe was used to reproducibly detect 1361 lipid features. Separation of lipids was performed using a reverse phase ACQUITY UPLC BEH C8 Column (2.1 × 100 mm, 1.7 μm, Waters co., Milford, MA, USA) coupled to a Vanguard precolumn. Mass spectra were recorded in both positive and negative modes. Spectra were analyzed with the XCMS software [50], which employed the “centWave” method for peak detection [13]. Of 1361 lipid features, 394 were lipid species annotated using an in-house library belonging to 16 different lipid classes, including triacylglyceride (TAG), acylcarnitine (CAR), phosphatidylcholine (PC), phosphatidylcholine plasmalogen (PC-P), ceramide (Cer), phosphatidylethanolamine (PE), phosphatidylethanolamine plasmalogen (PE-P), fatty acid (FA), sphingomyelin (SM), plasmanylphosphatidylcholine (PC-O), cholesteryl ester (CE), diacylglycerol (DAG), lysophosphatidylcholine (LPC), lysophosphatidylcholine plasmalogen (LPC-P), lysoplasmanylphosphatidylcholine (LPC-O), and lysophosphatidylethanolamine (LPE). After excluding all lipid species known to be altered by fasting status (*n* = 30; of which 26 were fatty acids) [51], 364 annotated lipids species not affected by fasting status remained and were carried forward to the analysis (Supplementary tables S1–S3).

### Genotyping and PRS calculation

Individuals were genotyped using the Illumina Infinium Global Screening Array-24 Kit (GSA Array, version 1 and 3; Illumina, San Diego, CA). Quality control and imputation (HRC [Version r1.1 2016] reference panel) were carried out following a pipeline described elsewhere [52]. In order to calculate the PRS-SCZ, PRS-BD, PRS-EF, and PRS-EA, we used the findings of the latest GWAS in SCZ [53], BD [54], executive function [55] and educational attainment [56] as discovery datasets. We calculated PRSs using the PRS Continuous Shrinkage approach (PRS-CS; “auto” settings) [57], to infer posterior SNP effect sizes under continuous shrinkage priors and eventually providing an individual estimate of the PRS-SCZ, PRS-BD, PRS-EF, and PRS-EA. PLINK 1.9 [58] was used for the final PRS scoring by summing the weighted effect generated by PRS-CS of each SNP that contributed to the PRS.

### Statistical analysis

Inverse normal- and log2-transformation were used for normalization of cognitive tests results and lipid levels, respectively and standardization was performed on both. A linear regression model in R version 4.3.0 (https://www.R-project.org/) was used to test for an association between lipid levels and cognitive tests results, and to check the effect of the PRS-SCZ, PRS-BD, PRS-EF, and PRS-EA on lipid levels. The class-enrichment analysis from LipidR package 2.15.1 [59] was used in R to test a two group comparison. Briefly, in this analysis lipids were classified based on their annotations, and enrichment scores and significance were determined for each lipid set using a permutation algorithm to indicate whether lipid classes were up- or downregulated between two groups (here, mean-based dichotomous cognitive tests results: low versus high performance). A more detailed explanation of the methodology can be found elsewhere (https://www.lipidr.org; [59]). Covariates included age, sex, diagnosis, duration of illness, body mass index, educational status (only in cognition analyses), first two ancestry principal components (only in PRS analyses), and medication (i.e., number of antipsychotics, antidepressants, mood stabilizers, and tranquilizers taken by each individual at the time of sampling). Sensitivity analysis of the covariates (to quantitatively check the robustness of putative causal estimates [60, 61]) in our study using “sensemakr” package in R has been conducted. We also re-ran different models of the regression analysis (Without covariates, with covariates having robustness values (RV) greater than 20%, and with all covariates). False discovery rate (FDR) was applied to adjust for multiple comparisons and the results were considered statistically significant if the adjusted *p*-value was < 0.05.

## Results

After normalization and standardization of the lipid intensities and the cognitive tests results, 619 individuals (344 males, 275 females; age: 40.8 ± 14.5 years) remained in the analysis. The demographic and psychopathological information of study participants are presented in Table 2. In addition, the comparisons of test scores for six cognitive test across three different diagnoses in our study (SCZ, BD, and HC) are presented in supplementary figure 1.

**Table 2:** Demographic and psychopathological data of study participants.

	SCZ	BD	HC	Test
**Subjects (n)**	**187**	**240**	**192**	**–**
**Sex (%female)**	**30**	**45**	**57**	**SCZ vs BD: χ-squared** = **9.248;** ***p*-value** = **0.00235** **SCZ vs HC: χ-squared** = **25.541;** ***p*-value** = **4.33** **×** **10**^**−7**^ **BD vs HC: χ-squared** = **5.056;** ***p*-value** = **0.024**
**Inpatient status (%inpatient vs. outpatient)**	**57**	**32**	–	**χ-squared** = **25.053;** ***p*-value** = **5.58** **×** **10**^**−7**^
**Age (years, mean** **±** **SD)**	**38.9** ± **12.9**	**44.7** ± **13.4**	**37.8** ± **16**	**SCZ vs BD: F-value** = **19.98;** ***p*-value** = **1.01** **×** **10**^**−5**^ SCZ vs HC: F-value = 0.531; *p*-value = 0.467 **BD vs HC: F-value** = **23.24;** ***p*-value** = **1.98** **×** **10**^**−6**^
**Educational status (%professional vs. high-school level)**	**38**	**60**	**66**	**SCZ vs BD: χ-squared** = **19.14;** ***p*-value** = **1.21** **×** **10**^**−5**^ **SCZ vs HC: χ-squared** = **27.00 ;** ***p*-value** = **2.02** **×** **10**^**−7**^ BD vs HC: χ-squared= 1.00; *p*-value = 0.32
**BMI (kg/m**^**2**^**, mean** **±** **SD)**	**28.2** ± **6.1**	**28.3** ± **6.3**	**24.1** ± **4.4**	SCZ vs BD: F-value = 0.026; *p*-value = 0.872 **SCZ vs HC: F-value** = **54.17;** ***p*-value** = **1.19** **×** **10**^**−12**^ **BD vs HC: F-value** = **59.84;** ***p*-value** = **7.53** **×** **10**^**−14**^
**Duration of illness (years, mean** **±** **SD)**	**12.1** ± **10**	**11.8** ± **11.2**	–	SCZ vs BD: F-value = 0.085; *p*-value = 0.77
**PANSS_Positive (mean** **±** **SD)**	**13.5** ± **5.8**	**8.9** ± **2.8**	–	**SCZ vs BD: F-value** = **114.6;** ***p*-value** < **2** **×** **10**^**−16**^
**PANSS_Negative (mean** **±** **SD)**	**15.1** ± **6.3**	**9.9** ± **4.1**	–	**SCZ vs BD: F-value** = **106.5;** ***p*-value** < **2** **×** **10**^**−16**^
**PANSS_General (mean** **±** **SD)**	**28.3** ± **8.9**	**22.4** ± **6.6**	–	**SCZ vs BD: F-value** = **59.48;** ***p*-value** = **9.28** **×** **10**^**−14**^
**YMRS Sumscore (mean** **±** **SD)**	**3** ± **4.6**	**3.7** ± **5.9**	–	SCZ vs BD: F-value = 2.191; *p*-value = 0.14
**IDS-C**_**30**_ **Sumscore (mean** **±** **SD)**	**14.9** ± **10.1**	**12.1** ± **10.1**	–	**SCZ vs BD: F-value** = **7.049;** ***p*-value** = **0.00826**

*BMI* body mass index, *PANSS* positive and negative syndrome scale, *YMRS* young mania rating scale, *IDS-C30* inventory of depressive symptomatology, *SCZ* Schizophrenia, *BD* bipolar disorder, *HC* healthy control.

**Fig. 1:**
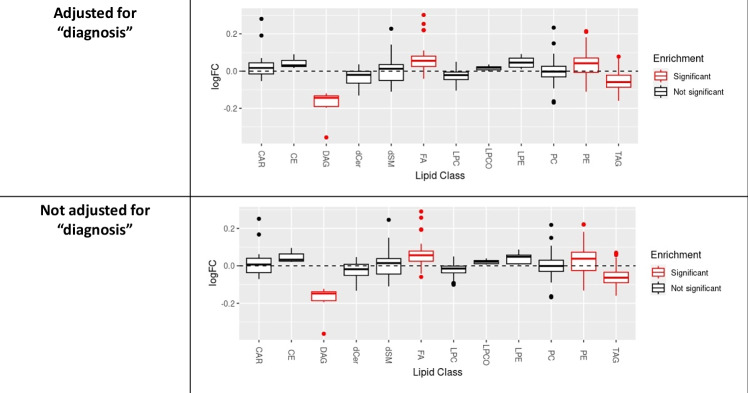
No major effect of mental health diagnosis (schizophrenia, bipolar disorder, healthy control) as a covariate in regression model on significance of PE class for the DG-SYM test in lipid class-based enrichment analysis in lipidR. Distribution of log fold change (logFC) per lipid class, with significantly enriched classes (marked in red) for mean-based low (as seen in the plots) versus high results in the dataset including all individuals with schizophrenia, bipolar disorder, and healthy controls (sample size: 531 individuals). *PE: phosphatidylethanolamine; DG-SYM: Digit-Symbol test*.

**Fig. 2:**
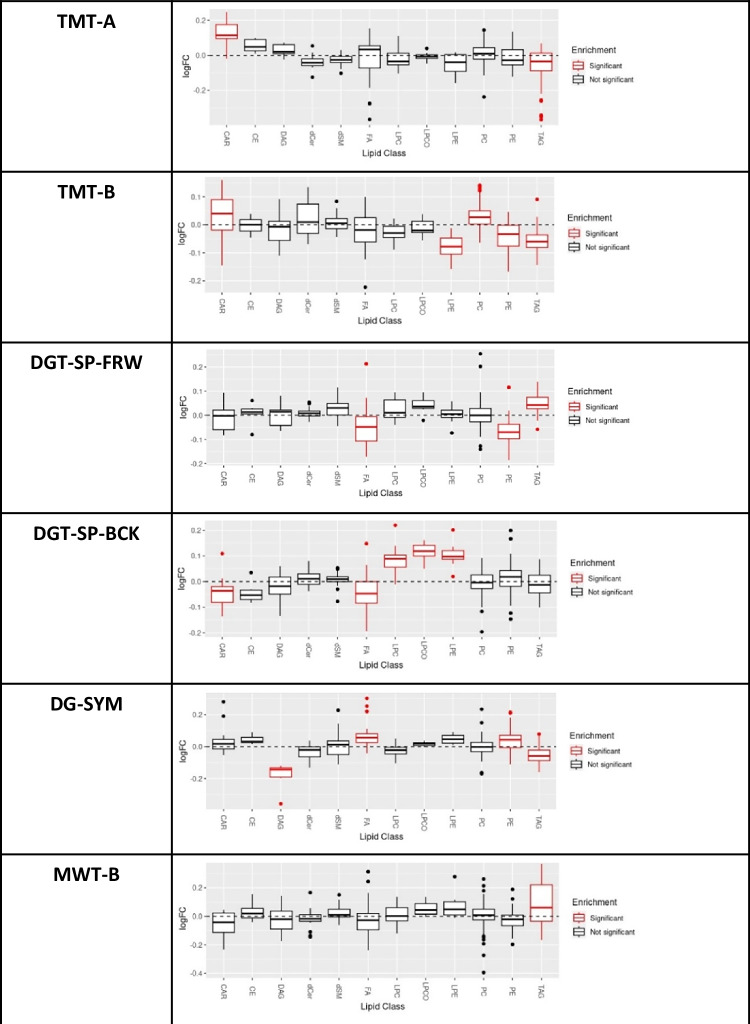
Lipid class-based enrichment analysis in LipidR for different cognitive tests. Distribution of log fold change (logFC) per lipid class, with significantly enriched classes (marked in red) for mean-based low (as seen in the plots) versus high cognitive tests results in the full dataset with the effect of covariates. *TMT-A, Trail-Making Test part A; TMT-B, Trail-Making Test part B; DGT-SP-FRW, Verbal Digit Span forward; DGT-SP-BCK, Verbal Digit Span backward; DG-SYM, Digit-Symbol; MWT-B, Multiple-choice Vocabulary Intelligence*.

Linear regression analysis indicated four individual lipid species to be significantly and negatively associated with DG-SYM test results: PE-P 42:5 (β = −0.134, FDR-adjusted p value = 0.039), PE-P 40:4 (β = −0.124, FDR-adjusted p value = 0.039), PE-P 40:5 (β = −0.125, FDR-adjusted p value = 0.042) belonging to the PE-P class and Cer 38:1 (β = −0.137, FDR-adjusted p value = 0.039) belonging to the Cer class. No statistically significant associations between individual lipid species (*n* = 364) and results of the TMT-A, the TMT-B, the DGT-SP-FRW, the DGT-SP-BCK, or the MWT-B could be identified (Supplementary table S4). Sensitivity analyses for age, sex, diagnosis, duration of illness, body mass index, educational status and medication use indicated that among these covariates, age with an RV of 0.34 and a R2Y ~ D|X of 0.15 had a relatively robust effect on DG-SYM test performance analyzed in this study (Supplementary Table S5, Figure S2). Running different models of linear regression analyses with different covariates yielded no change in our significant results; output of these models showed our four significant lipids remained unaffected (Supplementary Table S6). In addition to findings from linear regression analysis using normalized and standardized DG-SYM test results, the association between PE class and the dichotomous DG-SYM test results (mean-based low versus high) was also observed (adjusted *p*-value = 0.001) in the lipid class-based enrichment analysis using LipidR, which results in a list of significantly changes lipid classes. The presence or absence of a mental health diagnosis did not have a major impact on this outcome (Fig. 1). Furthermore, although single lipid species from the other lipid classes did not reach statistical significance in the linear regression model, DAG, FA, and TAG as lipid classes were also significantly associated with DG-SYM test performance in lipid class-based enrichment analysis using LipidR (Fig. 1, Fig. 2 and Supplementary table S7). This enrichment analysis also showed significant associations between other lipid classes and various cognitive tests: TAG and CAR with TMT-A; LPE, TAG, PE, PC and CAR with TMT-B; PE, FA and TAG with DGT-SP-FRW; LPC-O, LPE, LPC, CAR and FA with DGT-SP-BCK; and TAG with MWT-B (Fig. 2 and Supplementary table S5). Number of participants for each test in class-enrichment analysis is shown in supplementary table S8.

When PRS-SCZ, PRS-BD, PRS-EF, or PRS-EA were included in the linear models to check for the effect of genetic burden on the annotated lipid levels, nominal association was detected for several lipids for all PRSs. However, after FDR correction, no association remained significant *(*Supplementary tables S9–S12*)*, arguing against common genetic factors implicated in mental health disorders or educational attainment driving the observed associations. In this vein, we also checked the effect of PRS-SCZ, PRS-BD, PRS-EF, and PRS-EA on cognitive test results; that PRS-EF as expected was associated with most of the tests, such as DG-SYM test, and PRS-EA with DGT-SP-BCK and MWT-B tests (as previously also reported by our group [40]), but the results for PRS-SCZ and PRS-BD revealed no significant relationship between these PRSs and DG-SYM or other cognitive tests *(*Supplementary tables S13–S16*)*.

## Discussion

Lipidomic changes have previously been linked to both mental health disorders, such as SCZ or BD, and cognitive performance. In order to better understand the role of the lipidome in the context of deep cognitive phenotypes across the affective to psychotic spectrum, we capitalized on a large and cognitively deeply phenotyped study of individuals with mental health disorders with a rich plasma lipidomic dataset. We aimed to answered the question, whether specific lipid species or classes were associated with differential performance across various cognitive domains, including psychomotor and processing speed, executive function, short-term and working memory and crystalized intelligence and whether these associations were affected by diagnoses or genetic predispositions for mental health disorders or educational attainment.

In our hypothesis-free, untargeted lipidomics approach, lipids belonging to the PE-P class emerged as the main lipid class associated negatively with DG-SYM test performance, representative of processing and psychomotor speed. Our findings showed that higher levels of PE-P 42:5, PE-P 40:4, and PE-P 40:5 in plasma samples of our study are significantly associated with poorer DG-SYM test performance. The DG-SYM test mainly measures processing speed [41], the amount of time required to complete a series of cognitive tasks [62]. The lipid class-based enrichment analysis in LipidR validated the significant association of increases across the entire PE class with decreased processing speed as measured by the DG-SYM (Fig. 2). In addition, this analysis showed significant association of PE class with executive function (TMT-B) and short-term memory (DGT-SP-FRW) as different cognitive domains (Fig. 2).

Plasmalogens (-P) are a subclass of glycerophospholipids belonging either to the PC or PE class. Together with other phospholipids, they form lipid bilayer membranes. Plasmalogens makeup around 65% of all PEs and are present in all mammalian cells, but are especially numerous in neurons, cardiac muscle, and skeletal muscle [63, 64]. Plasmalogens have shown potential benefits on cognitive performance and their alterations can cause changes in ion channel and receptor function as well as a loss of membrane fluidity, myelination and myelin structure; processes of likely importance to cognitive function and the pathophysiology of mental health disorders, such as SCZ and BD [64–67]. Rare disorders of plasmalogen synthesis, such as rhizomelic chondrodysplasia punctata, lead to severe delays in motor development and intellectual disability [65, 68] and involve an impaired Schwann cell differentiation and consequent changes in synaptic neurotransmission, neuronal signaling and apoptosis and neuroinflammation, which may finally have an impact on cognitive performance [65, 66].

Our findings from the mean-based dichotomous analysis indicated that high peripheral levels of plasmalogens are related to poorer processing speed (DG-SYM) and executive function (TMT-B) but better short-term memory (DGT-SP-FRW), suggesting an intricate relation between plasmalogens and different cognitive parameters that warrants further investigation. As demonstrated by the lipid-species-based analyses, it is possible that individual lipid species or closely related groups of lipid species within a lipid class could have differential effects in the contexts of different cognitive functions. These differential effects could be lost in crude class-based enrichment analysis. The recent finding of a specific PE species (PE 18:1/20:4) as a potential predictor of overall cognitive decline measured using a composite score reflecting six cognitive domains in healthy aging individuals further speaks to this intricate interplay [69].

Although it is generally believed that it is difficult for plasmalogens to cross the blood-brain-barrier, recent evidence shows that in mice gastric uptake of plasmalogens has a direct effect on synaptic function and neuroinflammation in the murine brain [70]. Taken together, it is possible that the changes in plasma PE levels associated with DG-SYM results, could be reflective of alterations in brain cells membrane and myelin structure or function that are linked to altered processing speed.

Our regression analysis results also indicated a significant negative association between a ceramide species (C38:1 from Cer class) and processing speed. Higher plasma levels of C38:1 were associated with poorer DG-SYM test performance. Ceramides are a heterogeneous class of sphingolipids [71]. They are integral components of cell membranes and bioactive lipids involved in a variety of cell signaling pathways, including cell proliferation, differentiation, senescence, apoptosis, cell cycle arrest, inflammation, and responses to stress [72, 73]. Ceramides are one of the most abundant lipid classes in myelin sheaths, but disruption in their balance can represent an endogenous neurotoxin [8]. Moreover, in individuals with coronary artery disease, circulating ceramides have been related to changes in verbal memory and levels of some Cer species have also been linked to MDD [74–77]. Cer as a class in our lipidR analysis, however, were not linked to any measures of cognitive performance. Instead, at lipid class level, other interesting findings emerged. For example, working memory, as measured by the DGT-SP-BCK, was associated with levels of CAR and all three lysophospholipid classes (LPC, LPC-O, and LPE). Lysophospholipids have near ubiquitous functions in membrane shaping, cell trafficking, cell growth and death, and inflammatory cascades and are closely related to lysophosphatidic acid (LPA) [78]. LPA and LPA receptor signaling pathway deficits have been linked to different types of memory in both mice and zebrafish [79–81], providing a hypothetical biological link for the observed association.

In our recent multi-cohort case-control study (3 cohorts with varied cultural and demographic backgrounds including the PsyCourse Study), a profile of 77 significant and reproducible lipid species, including ones belonging to the PE-P and Cer classes, were found to be associated with SCZ; however, overlapping changes were also observed in BD and MDD [13]. As far as we know, our current study represents first evidence at lipidome-wide scale, that peripheral lipid particularly plasmalogens could reflect cognitive performance, most concretely for psychomotor processing speed, both in individuals with mental health disorders and those without.

Age in our study had a relatively robust effect on processing and psychomotor speed of cognitive function, while medications showed relatively low robustness values as demonstrated by sensitivity analysis (Supplementary table S5). Nevertheless, the significant findings of our study remained stable across different models, including those that included or did not include the covariates such as age and four groups of medications (Supplementary table S6).

While overall PRS-SCZ, PRS-BD, PRS-EF, and PRS-EA were not related to lipid levels in our analysis, more refined analysis based on PRS for specific lipid species or lipid-pathway-specific PRS would be a next interesting analytic step. However, comprehensive lipid-metabolism-based PRS analysis would likely require larger sample sizes not currently available for the phenotypes of interest to this study. In this context of PRS, common genetic variants in retinoid signaling (a lipid metabolism-related pathway) genes have been shown to be associated with the severity of cognitive impairment in individuals with SCZ [82]. This highlights the potential utility of our results and those of others in unraveling the complex interplay between genetics, lipid profiles, and cognitive performance, thus paving the way towards predictive risk models.

Nonetheless, our study has several limitations. Even though obtained in one of the currently largest studies of individuals with untargeted lipidomics data and in-depth cognitive performance metrics, our results would benefit from replication in an independent dataset in the future. One of our study’s main limitations is that we were only able to examine peripheral lipidomic profiles. Lipidomic analysis of cerebrospinal fluid samples could be more informative in understanding which lipids are linked with cognitive performance, although the current paradigm of cerebrospinal fluid as the gold standard biomaterial for cognition biomarkers is currently being challenged and potentially rewritten for neurodegenerative diseases [83]. Study recruitment in a naturalistic setting also adds different clinical states and differing treatment regimes for each study participant at the time of evaluation as additional challenges. These were addressed by including gross medication categories and disease duration as covariates but confounding cannot be ruled out entirely. More granular information on, for example, cognitive training that study participants could potentially have received also is not available but could, of course, have an impact on cognitive testing performance. When a very conservative overall correction was applied to the regression model results for all six cognitive tests together, none of the lipid-species-significant findings remained significant. Lastly, no differential relationship between plasma lipidomic profiles and cognitive performance could be identified in individuals with SCZ and BD as opposed to those without mental health disorders, therefore potentially limiting the utility of our findings for future predictive strategies specific to the cognitive dysfunction affecting disease course and outcome in SCZ and BD.

In conclusion, we found here the negative association of PE lipid class with DG-SYM test performance, which represents the processing and psychomotor speed. While it is becoming increasingly clear that dysregulated blood lipid profiles are present in individuals with major mental health disorders, their extent and links to the etiopathology and phenotypic presentation are only partially understood. Although speculative at this point, links between lipidomic profiles and cognitive function could exist along the plasma-to-brain axis. Such a link would be the basis necessary for studies focusing on clinical translation and drug development and repurposing in order to address the need to ameliorate the outcome-determining cognitive dysfunction in individuals with mental health disorders.

## Supplementary information

Supplementary Figures S1_S2

Supplementary Tables S1-S16

## Data Availability

A unique feature of the PsyCourse Study is that it has been conceptualized as a continuously growing data resource available to the scientific community. Data sharing will be based on mutually agreed research proposals and within the Open Science framework of the PsyCourse Study (see psycourse.de/openscience-en.html).
