# Supplementary material for: Investigating the association of the plasma lipidomic profile with cognitive performance and genetic risk in the PsyCourse study
Source: Transl Psychiatry. 2025 Mar 28;15:105. doi: 10.1038/s41398-025-03323-5 (PMC11953450; doi:10.1038/s41398-025-03323-5)
Supplement: Supplementary file 1 — Supplementary Figures S1_S2 [file 41398_2025_3323_MOESM1_ESM.docx]

***Supplementary Figures***

**Investigating the association of the plasma lipidomic profile with cognitive performance and genetic risk in the PsyCourse Study**

| **TMT-A** | 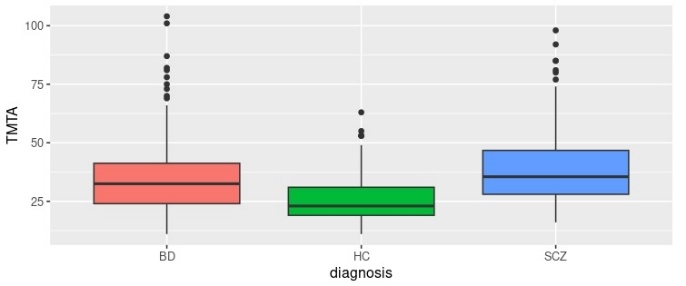 |
| --- | --- |
| **TMT-B** | 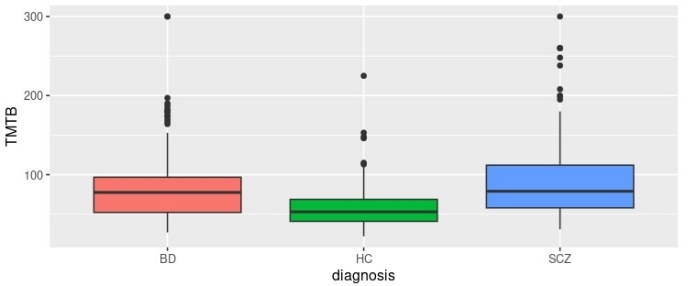 |
| **DGT-SP-FRW** | 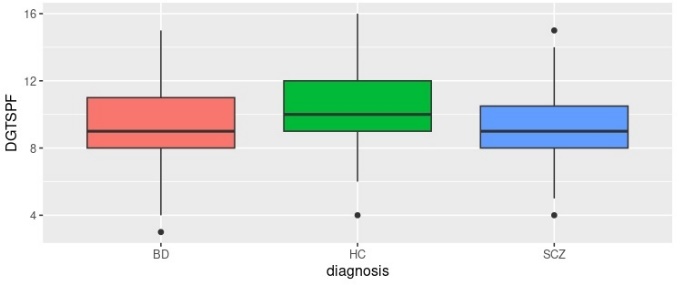 |
| **DGT-SP-BCK** | 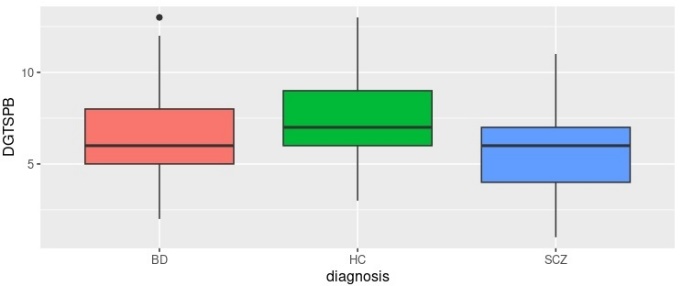 |
| **DG-SYM** | 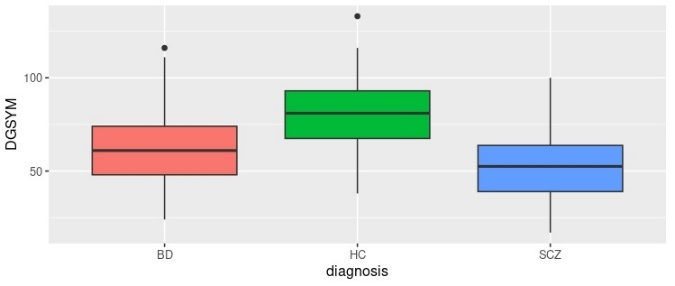 |
| **MWT-B** | 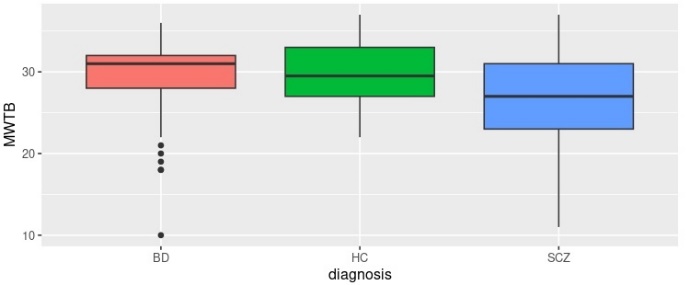 |

**Figure S1.** **Comparison of test scores for each cognitive test across different diagnoses (SCZ, BD, and HC).** *SCZ, schizophrenia; BD, bipolar disorder; HC, healthy control*

| **Age** | **Sex** |
| --- | --- |
| 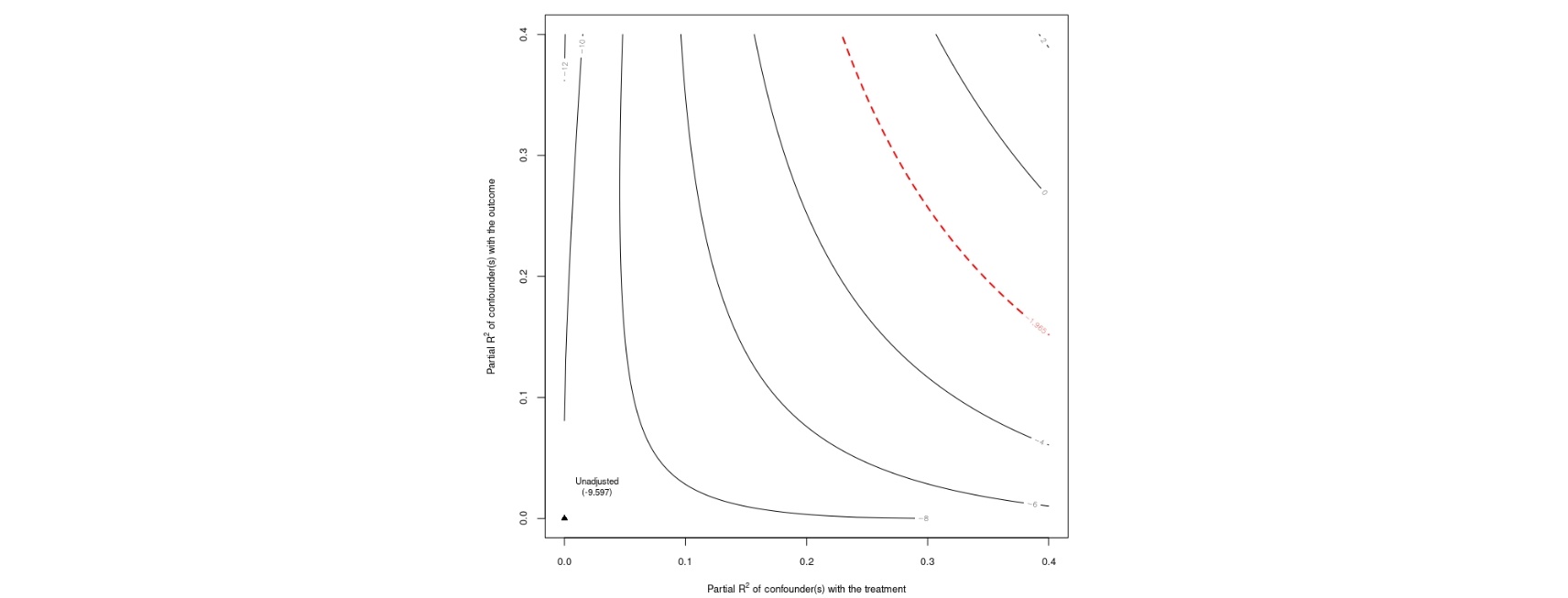 | 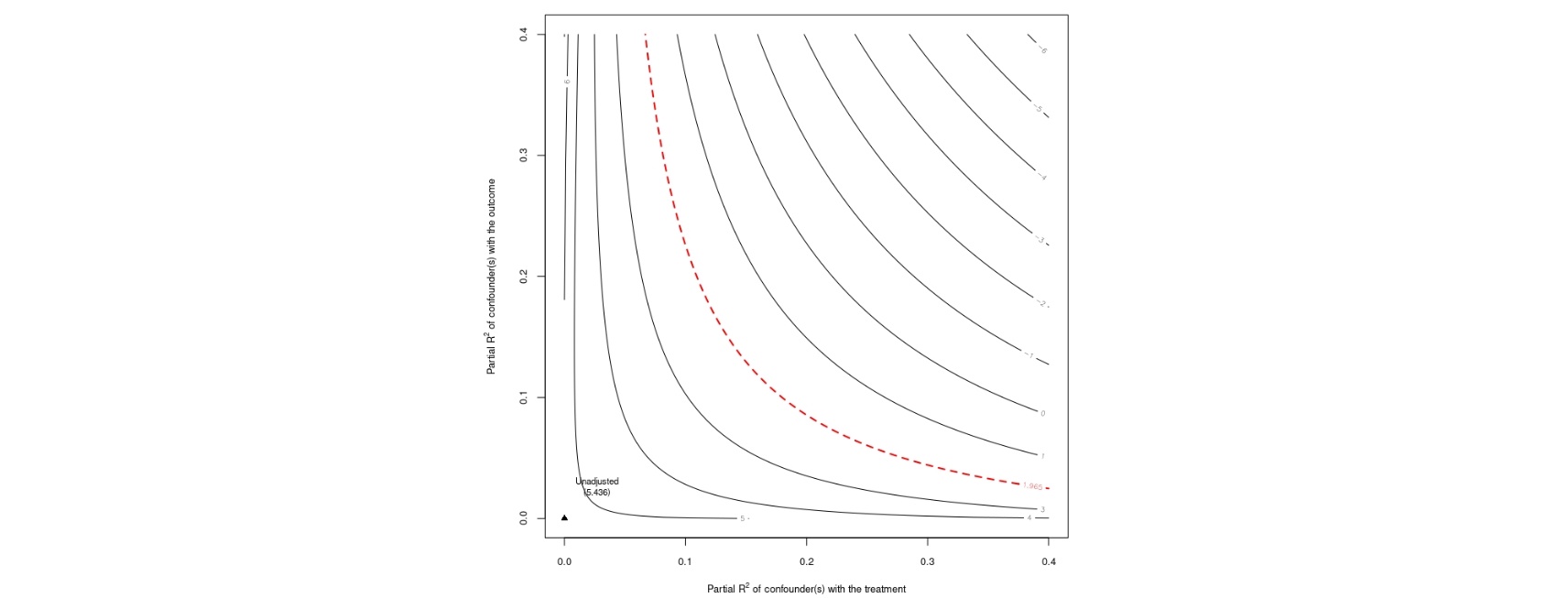 |
| **Diagnosis** | **Educational status** |
| 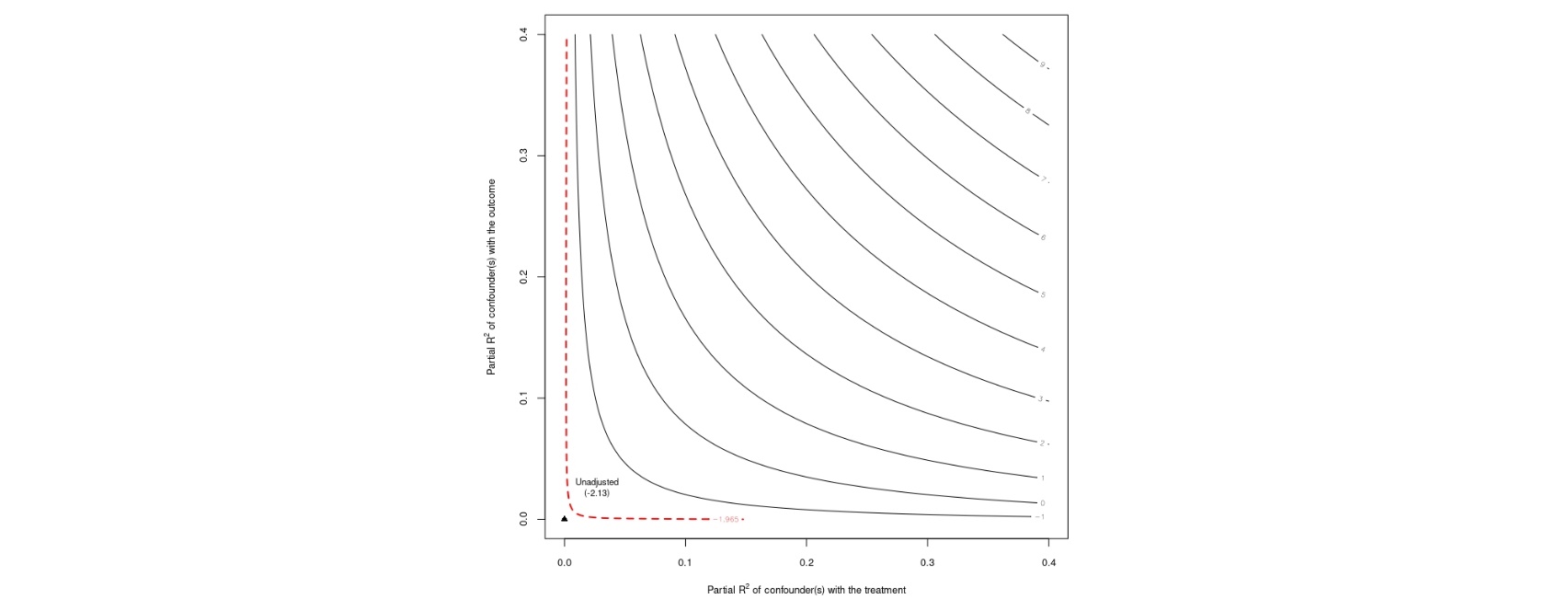 | 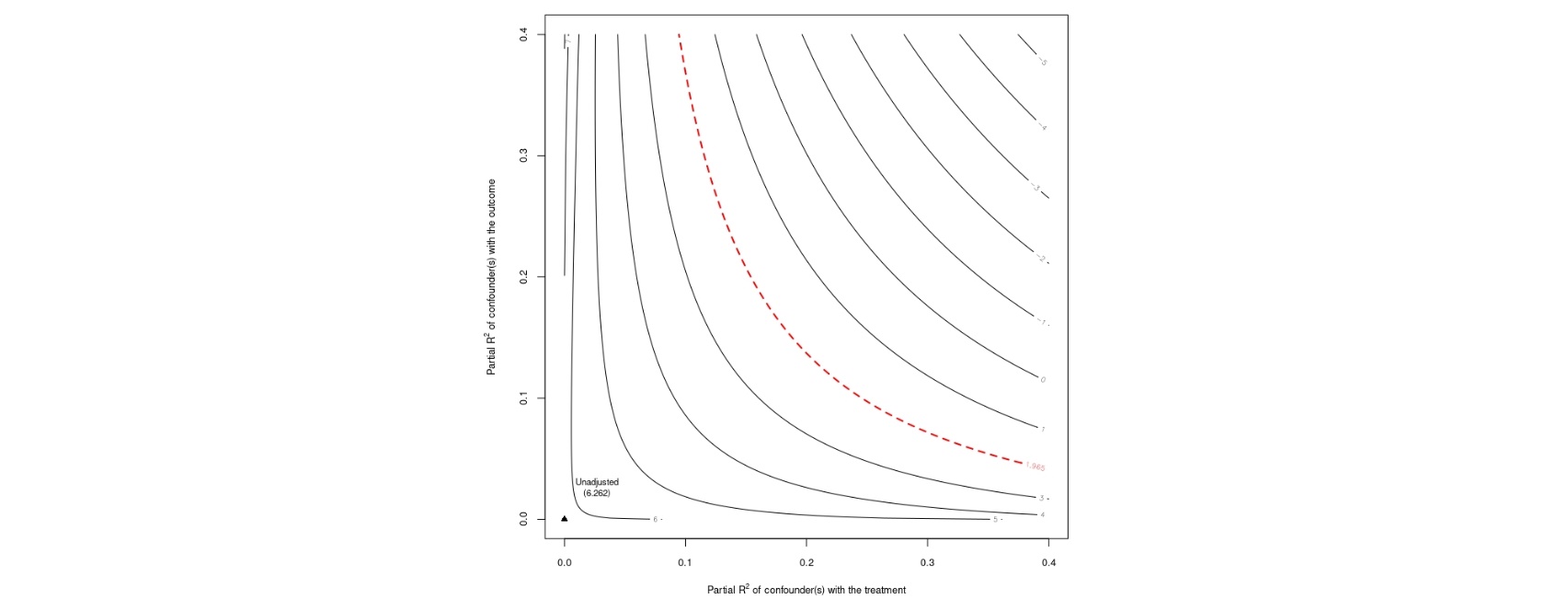 |
| **Duration of illness** | **BMI** |
| 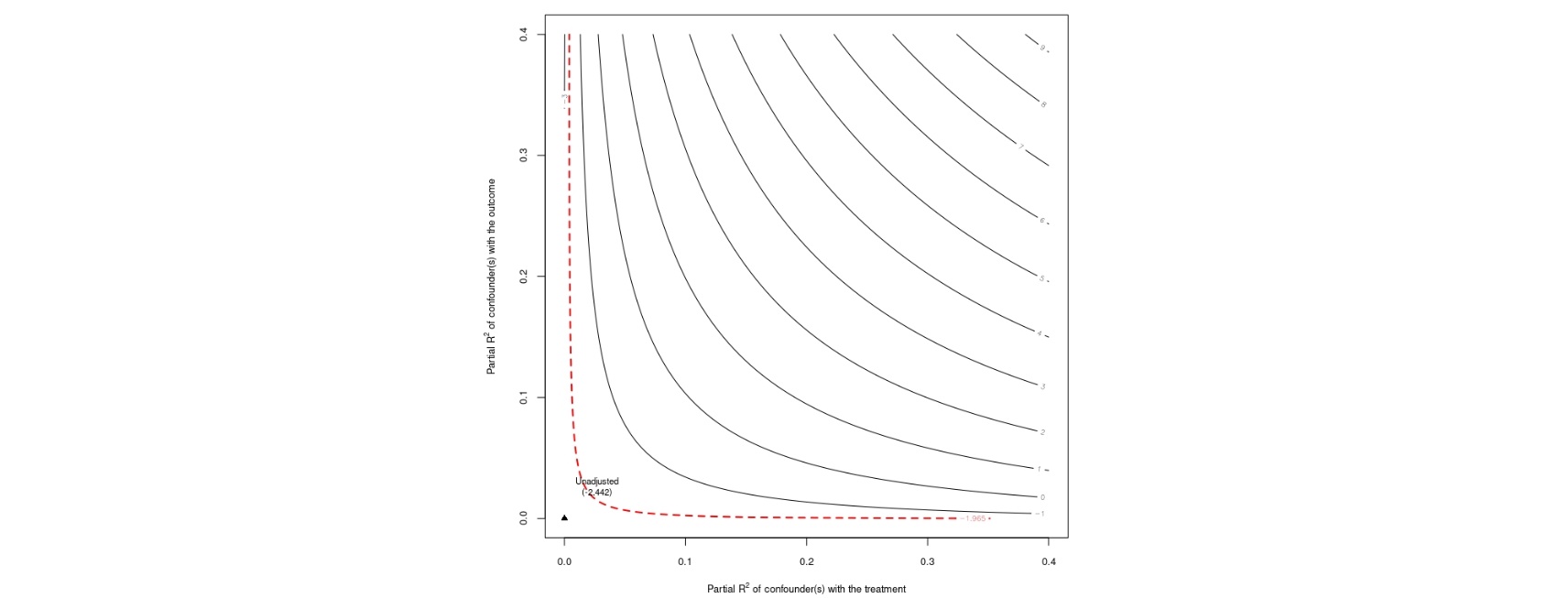 | 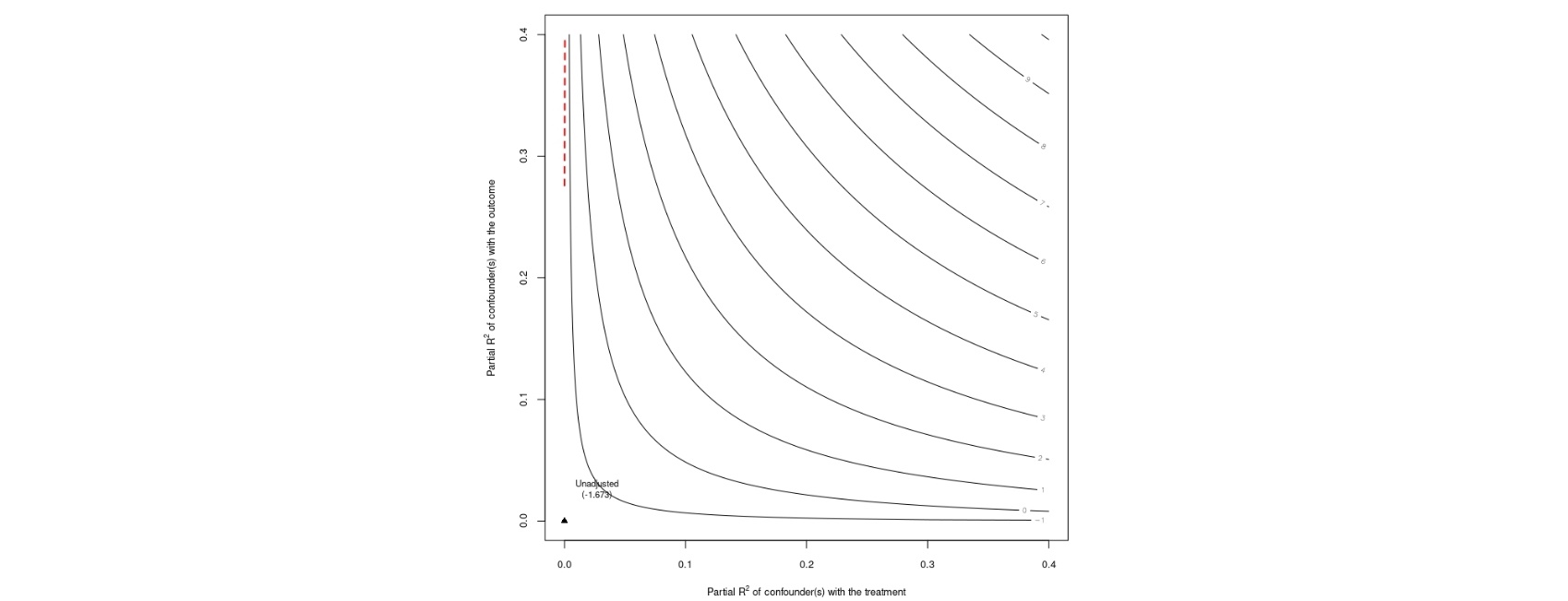 |
| **Antipsychotics** | **Antidepressants** |
| 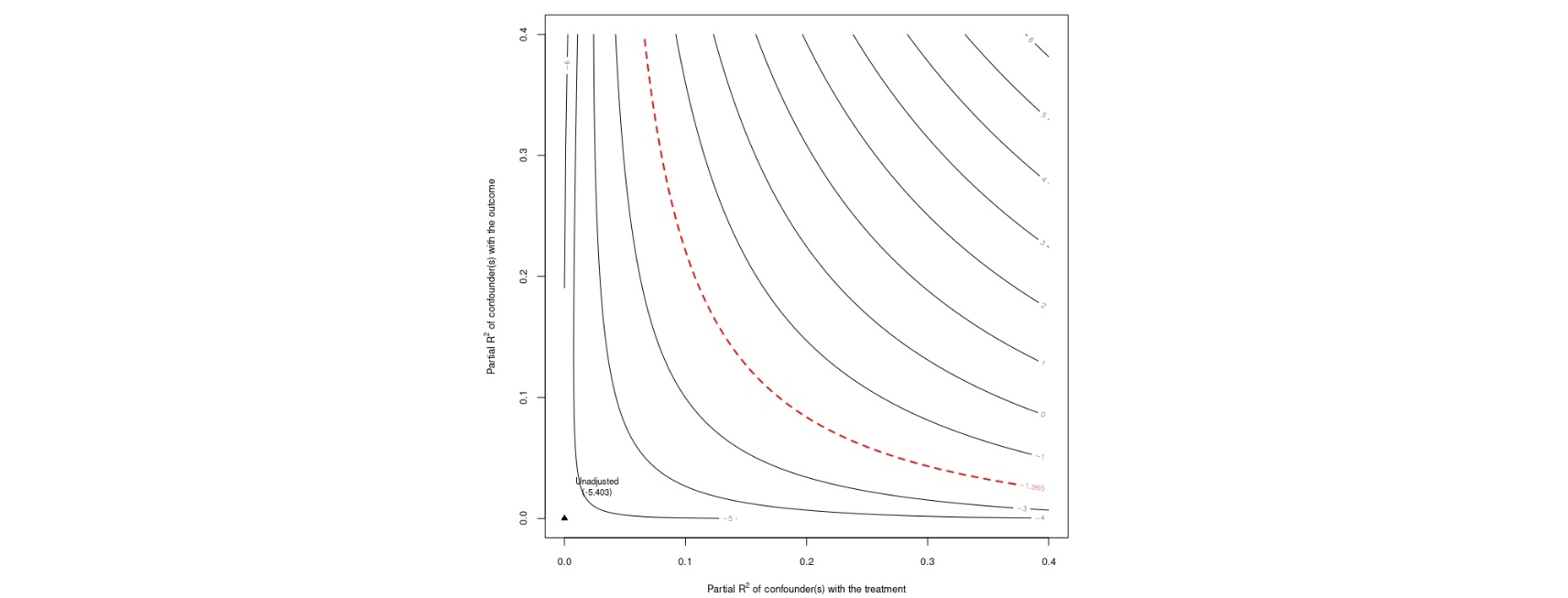 | 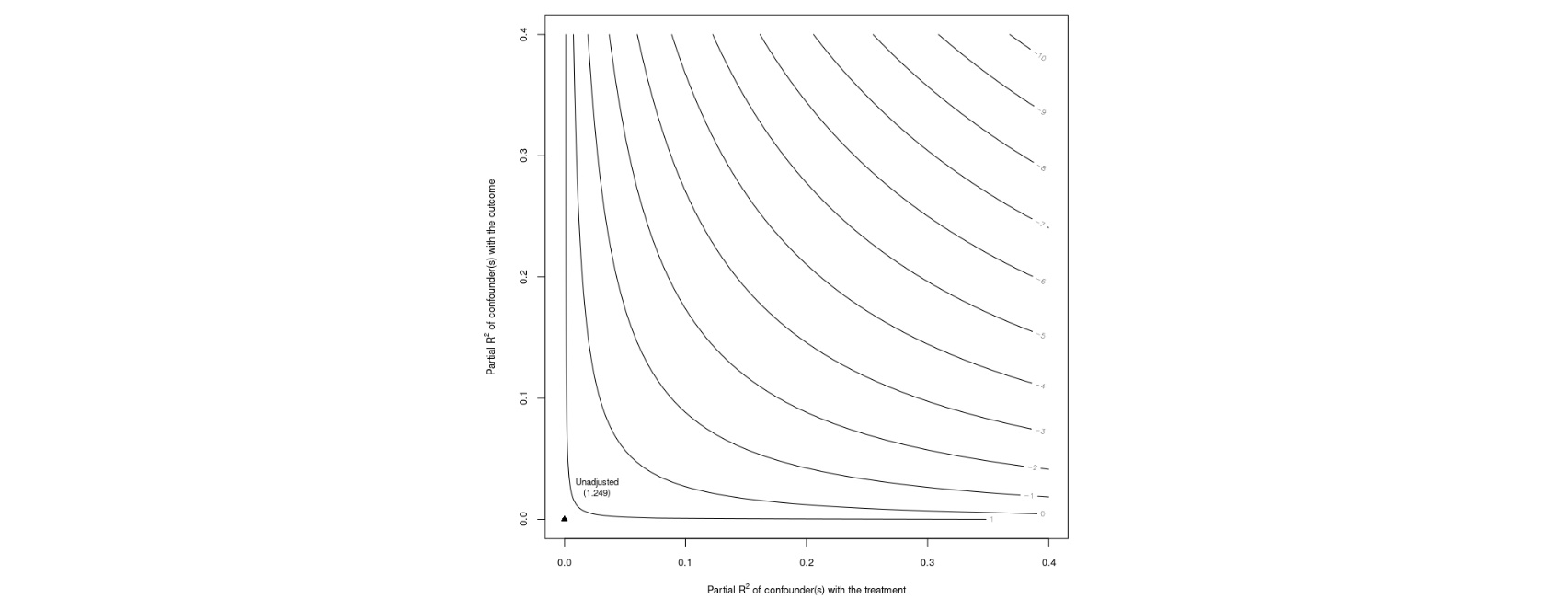 |
| **Mood stabilizers** | **Tranquilizers** |
| 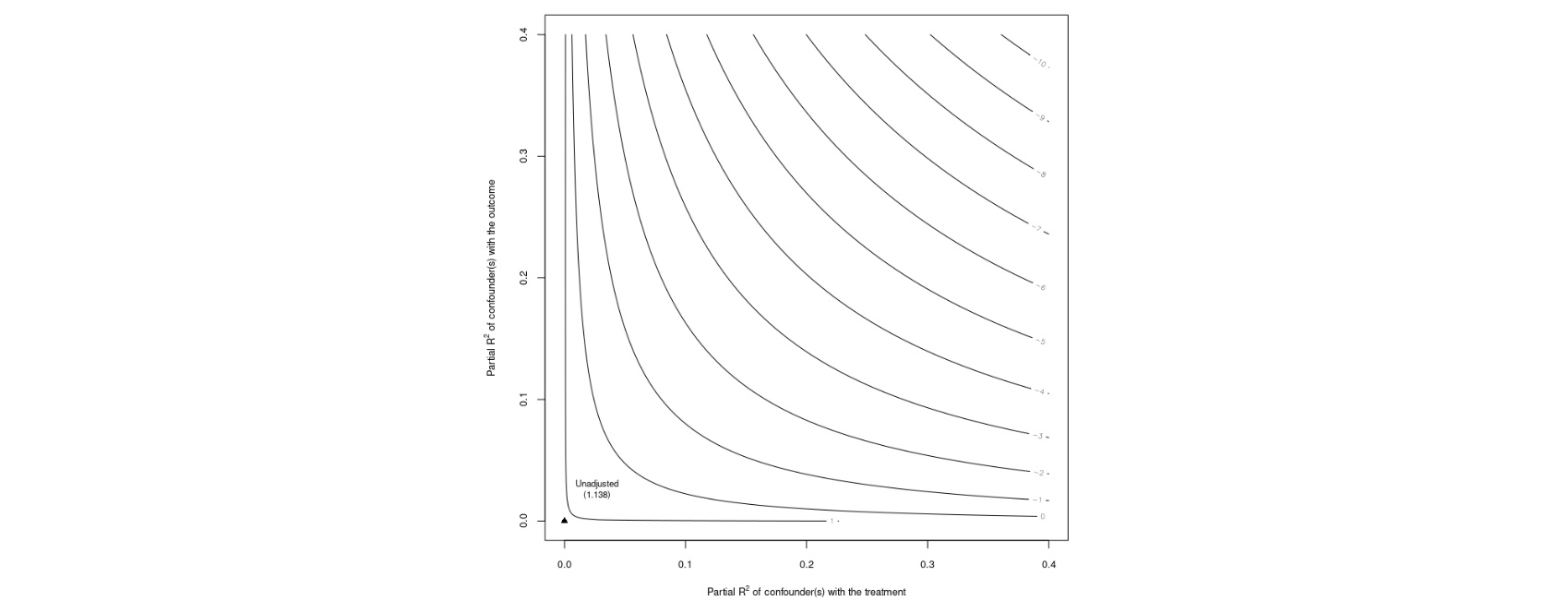 | 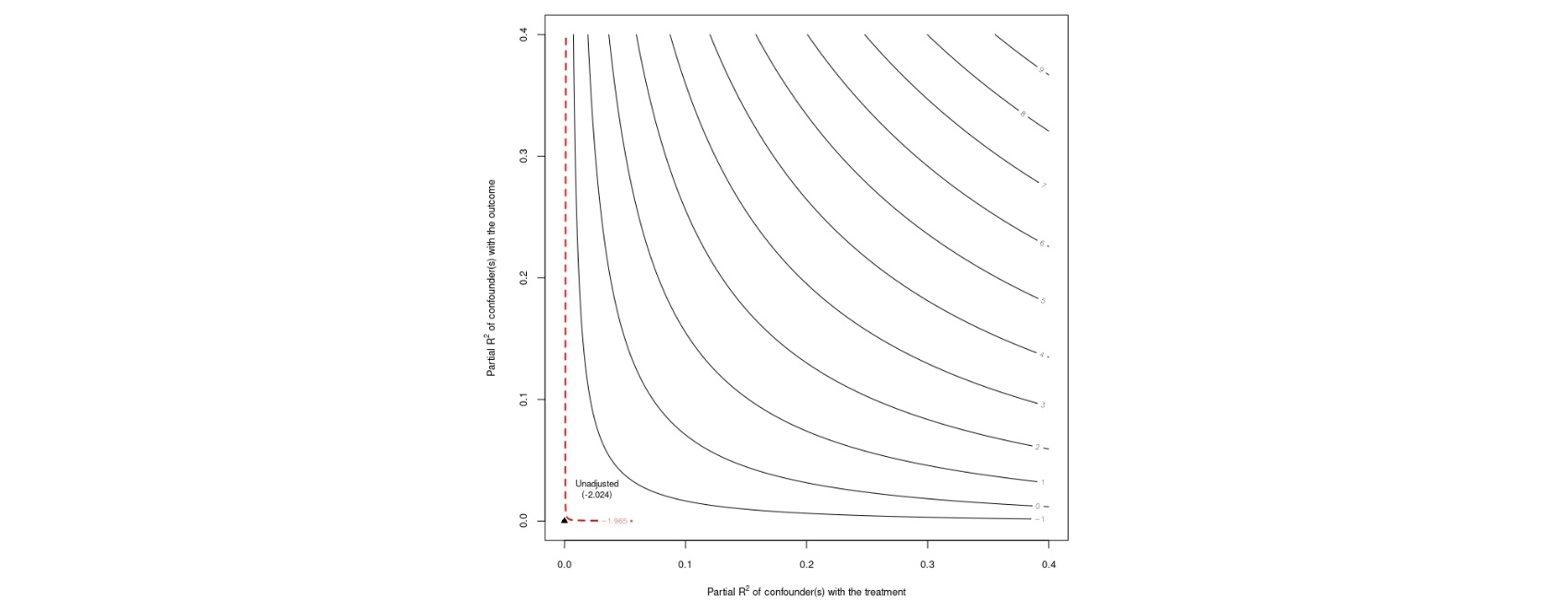 |

**Figure S2. Sensitivity analysis for the covariates in our study**. The plot reveals examining the sensitivity of the *t-value* for testing the null hypothesis of zero effect.

Verbal interpretation of sensitivity statistics^1^, for example for “Age”:

Unadjusted Estimates of “age”:

Coef. estimate: -0.0248; Standard Error: 0.0026; t-value (H0:tau = 0): -9.5967

Sensitivity Statistics:

Partial R2 of treatment with outcome: 0.1505; Robustness Value, q = 1: 0.3415; Robustness Value, q = 1, alpha = 0.05: 0.2833

Partial R2 of the treatment with the outcome: an extreme confounder (orthogonal to the covariates) that explains 100% of the residual variance of the outcome, would need to explain at least 15.05% of the residual variance of the treatment to fully account for the observed estimated effect.

Robustness Value, q = 1: unobserved confounders (orthogonal to the covariates) that explain more than 34.15% of the residual variance of both the treatment and the outcome are strong enough to bring the point estimate to 0 (a bias of 100% of the original estimate). Conversely, unobserved confounders that do not explain more than 34.15% of the residual variance of both the treatment and the outcome are not strong enough to bring the point estimate to 0.

Robustness Value, q = 1, alpha = 0.05: unobserved confounders (orthogonal to the covariates) that explain more than 28.33% of the residual variance of both the treatment and the outcome are strong enough to bring the estimate to a range where it is no longer 'statistically different' from 0 (a bias of 100% of the original estimate), at the significance level of alpha = 0.05. Conversely, unobserved confounders that do not explain more than 28.33% of the residual variance of both the treatment and the outcome are not strong enough to bring the estimate to a range where it is no longer 'statistically different' from 0, at the significance level of alpha = 0.05.

The treatment effect of the covariate can handle strong confounders that explain almost all of the residual variation in the treatment and the outcome when the robustness value of the covariate is close to 1^2^.

***References***

1 R package sensemakr available on CRAN: https://cran.r-project.org/package=sensemakr. .

2 Cinelli C, Hazlett C. Making Sense of Sensitivity: Extending Omitted Variable Bias. *Journal of the Royal Statistical Society Series B: Statistical Methodology* 2020; **82**: 39–67.
